# The Effect of Silica Fume and Organosilane Addition on the Porosity of Cement Paste

**DOI:** 10.3390/molecules25081762

**Published:** 2020-04-11

**Authors:** Andrea Crețu, Carlos Mattea, Siegfried Stapf, Ioan Ardelean

**Affiliations:** 1 Fachgebiet Technische Physik II/Polymerphysik, Institute of Physics, Technische Universität Ilmenau, 98684 Ilmenau, Germany; carlos.mattea@tu-ilmenau.de; 2 Department of Physics and Chemistry, Technical University of Cluj-Napoca, RO-400114 Cluj-Napoca, Romania; ioan.ardelean@phys.utcluj.ro

**Keywords:** portland cement, silica fume, APTES, organosilane, porosity, ^1^H NMR

## Abstract

The present work systematically investigates the influence of silica fume and organosilane addition on the hydration dynamics and the capillary pore formation of a cement paste. The cement samples were prepared with two water-to-cement ratios with increasing amounts of silica fume and of (3-Aminopropyl)triethoxysilane (APTES) organosilane. Low-field ^1^H nuclear magnetic resonance (NMR) relaxation measurements were performed during the hydration of the samples and after hydration, in order to reveal the dynamics of water molecules and the pore distribution. Increasing concentrations of silica fume impact the perceived hydration dynamics through the addition of magnetic impurities to the pore solution. However, there is a systematic change in the capillary pore size distribution with an increase in silica fume concentration. The results also show that the addition of APTES majorly affects the hydration dynamics, by prolonging the dormancy and hardening stages. While it does not influence the pore size distribution of capillary pores, it prevents cyclohexane from saturating the capillary pores.

## 1. Introduction

For decades, cement has remained the most important material in the construction industry. Because of the high demand of energy and the large amount of carbon dioxide released into the atmosphere during its production, it has a high impact on the environment. Reducing the impact of cement industry on the environment requires an improvement of the mechanical properties of cement-based materials. Thus, if a lower quantity of cement is necessary for the same performance of a cement-based structure, the environmental impact is greatly reduced. This is an ongoing challenge that has started in the middle of the last century [1,2,3,4,5,6].

Improving such a complex material is not possible without understanding its chemistry and its responses to different factors. There have been several scientific approaches towards increasing the mechanical resistance of cement-based materials, especially by introducing complementary mineral or organic admixtures, both in solid and liquid state. Silica fume (SF) is such a mineral admixture that is used to improve the cement-based materials [7,8,9,10,11]. Its effect on the compressive and flexural strength of concrete has been well documented in numerous studies, all praising its pozzolanic reactivity [9,10,11,12,13,14,15], in which more calcium silicate hydrate (C-S-H) is produced when silica fume reacts with the calcium hydroxide (CH) in the material. It has been noted that adding silica fume to cement-based materials increases the density of C-S-H, which has an effect on both the compressive and flexural strengths and the diffusivity of chloride ions [16].

Silica fume is a pozzolanic admixture that has been used for producing high-performance concrete for more than four decades [17,18]. It is an industrial byproduct composed of agglomerations of silicon dioxide that condense from vapor into spherical particles with sizes that range between 100 nm and 1 μm, with some exceptions exceeding 10–50 μm [19]. Silica fume has been in use as an admixture, yet its effects on the final properties of the cement-based materials incorporating silica fume are not entirely elucidated. This is true, especially with regards to its presumed activity as a nucleation center for C-S-H in the hydration process [7,9] and the effects on capillary porosity [12,20] and the density of C-S-H.

Organosilanes are substances that interact with organic and inorganic materials, due to their structure that contains a silicon atom connected to four organofunctional groups. Usually, as is the case of (3-Aminopropyl)triethoxysilane (APTES), three of these groups hydrolyze in the presence of water and allow for the organosilane to attach to a surface through hydrogen bonds, or to polymerize with other identical molecules through Si-O-Si bonds. Both cement and silica fume are composed of silicon atoms and present -OH groups that are close to the surface, allowing for the hydrolyzed APTES to physically bond to their surfaces and later condense on the hydration products if the water is completely removed, through a process known as silanization [21].

APTES has been used previously in cement-based materials and it has been found to increase the workability of cement paste [20,22], to delay the hydration processes [20,22,23] and to increase flexural and compressive strength [20,22]. The increase in the strength can be attributed to a decrease in the pore size of the manufactured materials. In the present work we will systematically investigate their effects on cement paste mix prepared with two water-to-cement ratios using NMR relaxation measurements to clarify the contribution of silica fume and APTES to the hydration dynamics and pore size distribution.

The hydration of materials containing cement is a well-known process that consists of several stages or phases, in which the minerals that make up cement react with water over time. These and secondary reactions with or in the presence of admixtures produce a solid matrix of hydrated minerals with high porosity, consisting of several pore sizes, from nanopores that range in size from 2–10 nm (intra-C-S-H pores) to 50–600 nm (capillary pores). The hydration stages and the resulting porous structure have been presented in detail in previous works [8,21,23]. Admixtures influence the duration and intensity of different hydration stages and they can be monitored in a noninvasive manner while using NMR techniques [24,25,26]. The NMR methods provide information about the reactions which take place inside the material, the intensity or speed of these reactions, the hydration stage at a certain moment in time, the amount of free water (confined in capillary pores) or bound water (confined in smaller pores or physically bound between layers of C-S-H), the effect of a mineral admixture on the pore size distribution [8,21,27,28]. NMR methods also provide information regarding the interactions between the liquid molecules and the pore surface [8], about the particle surface, the hydration dynamics at different depths inside a hydrating cement paste [29], or the magnetic impurities that are released into the liquid as cement grains dissolve [26].

## 2. Results and Discussion

The same samples have been used for both experimental methods, first as fresh mixtures for monitoring the hydration process and then in their hardened state, after 28 days of hydration. The samples were prepared sequentially, while using the formulations that are indicated in Table 1.

**Figure 1:**
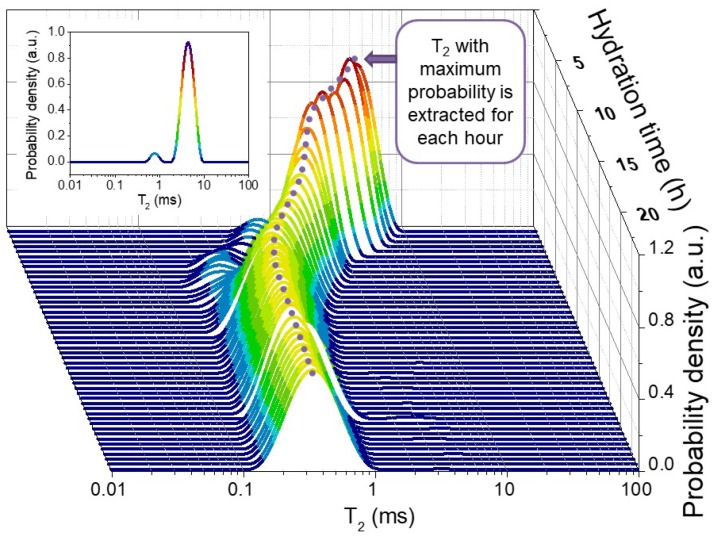
Evolution of the probability density of T_2_ during the first 30 h of hydration in the case of sample 400. Only T_2_ with the maximum probability is extracted for each hour. Inset: Probability density distribution of T_2_ values at 30 min. after mixing.

**Figure 2:**
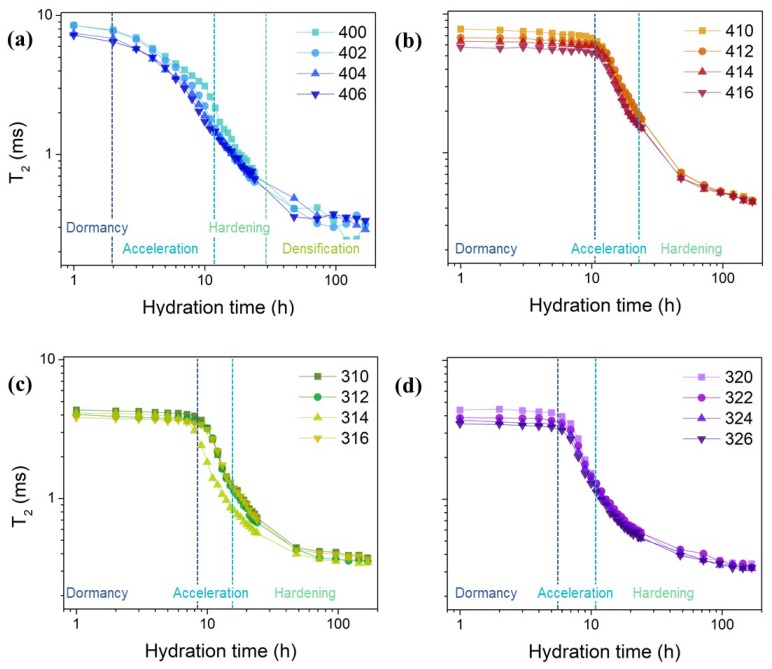
Evolution of the T_2_ value with the maximum probability for the indicated samples: (**a**) 400–406; (**b**) 410–416; (**c**) 310–316; and, (**d**) 320–326. The different stages of hydration are outlined for each set of samples.

The CPMG echo trains that were recorded during the hydration allow for the extraction of relaxation time distribution using a numerical inverse Laplace transform [30]. Figure 1 shows an example of such distribution for sample 400. One can observe two distinct relaxation components in the early hydration stages that merge in a broader distribution after the acceleration stage of hydration (6 h in the case of the sample in Figure 1). The main component at the beginning of hydration, arising at long relaxation times and with a larger area, slowly shifts towards shorter values as water from the capillary pores becomes chemically bound in C-S-H and physically confined between C-S-H layers, as demonstrated by the decrease in the peak area [28,31]. It is possible to extract the T_2_ values of the peak maximum, as there are no significant differences between the peak widths for these samples (see Figure 1). Figure 2 shows the T_2_ values extracted for the samples prepared with a water-to-cement ratio of 0.4 and 0.3, respectively. The obtained curves are representative for the hydration dynamics in the considered samples [23].

When comparing the samples in each set presented in Figure 2, a noticeable difference arises irrespective of the water-to-cement ratio or the organosilane concentration. A slight decrease of the T_2_ corresponding to water in capillary pores with increasing concentrations of silica fume is observed. The small reduction of the relaxation time by silica fume addition can be explained by the presence of paramagnetic impurities in their structure, which act as relaxation centers for water molecules, additionally contributing to the relaxation mechanism [32]. This enhancement of the relaxation rate by the presence of SF particles is apparent until the intra- and inter-C-S-H contributions to the signal can no longer be neglected [28]. The effect of SF particles on reducing the relaxation rate can be neglected when these latter contributions become dominant and as the SF particles start to react with the newly formed calcium hydroxide (CH).

Referring to the influence of APTES on the hydration dynamics, one can observe an expanding of the dormancy stage when the admixture is introduced in the cement paste mixture. The extent of the effect of APTES on dormancy stage duration seems to depend on the water-to-cement ratio and organosilane concentration. When no organosilane is added (Figure 2a), the hydration progresses normally, with no delay. The dormancy stage is completed at around 2 h from mixing, and then the T_2_ starts to decrease. The acceleration period follows, lasting up to 10 h from mixing. Hardening is noticeable between 10–30 h and then densification follows until the end of the experiment [33]. In the case of APTES addition, at the same water-to-cement ratio (Figure 2b), the dormancy extends up to 10 h. The acceleration progresses normally, up to 20 h after mixing, and then the hardening stage progresses slowly until the last measurement. The densification is not observed in the case of this set of samples, due to the expansion of the dormancy stage.

When decreasing the water-to-cement ratio, the extension of the dormancy stage is less obvious, even when increasing the amount of organosilane. For 1% APTES addition, there is an extension of the dormancy stage up to 8–9 h (Figure 2c), while, for 2% addition, the dormancy stage extends only to 5 h after mixing (Figure 2d). After the dormancy stage, the hydration progresses normally, reaching a stable point that can be considered to be the beginning of the densification stage. As the same effect is apparent when using superplasticizers, the currently accepted explanation of the extension of the dormancy is based on the formation of a layer of molecules on the surface of cement grains, based on the negative charges of the organic chains [34]. This layer slows down the dissolution process of the minerals and increases the time that is required for reaching the high ionic concentration necessary for the start of the acceleration period. This behavior has also been observed when adding superplasticizers to cement-based materials [35].

**Figure 3:**
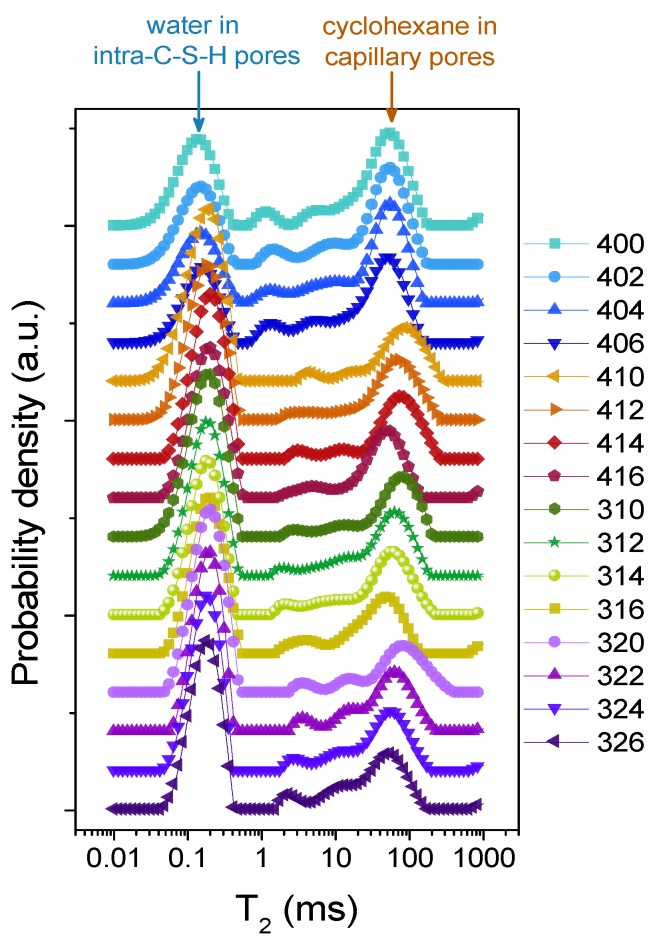
T_2_ distributions in all sets of samples. The samples were saturated with cyclohexane, after hydration.

As a non-polar liquid, cyclohexane has been used previously in exploring the pore structure of hydrated cement paste [28]. It was shown that it only fills the capillary and inter-C-S-H pores, which can be observed in the T_2_ distribution at longer times (from 2 ms up to 100 ms in Figure 3). After the main hydration reactions, there is only a slight difference between the shapes of the T_2_ distributions of the hydrated samples when filled with cyclohexane, although the hydration dynamics are modified by the addition of APTES (Figure 3). The water is not removed from the intra-C-S-H pores and it can be observed between values 0.05 and 0.5 ms on the T_2_ distribution [36] presented in Figure 3.

**Figure 4:**
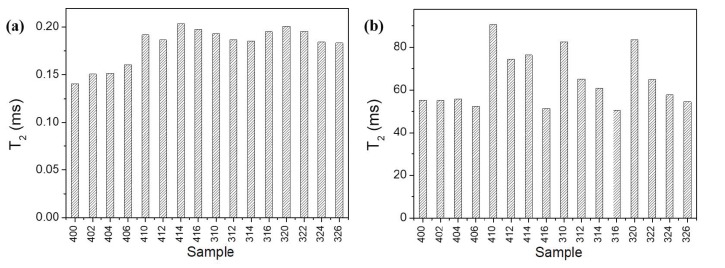
The position of the peak maximum in the case of intra-C-S-H water (**a**) and capillary cyclohexane (**b**).

In the case of the intra-C-S-H pores, in the group of samples containing APTES (samples 406 through 326), there is a shift of the maximum value of the peak towards slightly larger values—T_2_ of 0.15 ms in the original group (samples 400–406) and 0.2 ms in the group containing APTES, as can be observed in Figure 4a. This effect is consistent throughout the samples, irrespective of the presence of silica fume, which reacts with calcium hydroxide early during the hydration, producing more C-S-H, and it might be explained by the interference of the APTES with the usual hydration process, leading to the formation of larger intra-C-S-H pores where the APTES molecules are trapped.

Another aspect that is observed from the same experiment is the steady decrease in the T_2_ values of capillary pores, which are filled with cyclohexane, with an increase in silica fume concentration, but only in the presence of APTES (Figure 4b). This effect might be a manifestation of a decrease in the size of capillary pores, as the silica fume concentration increases. It might also lead to changes in mechanical properties, such as the flexural or compressive strength of the composites, which will be explored in further works.

The peak area is proportional to the amount of liquid confined inside each pore type. In the case of samples containing APTES, an interesting feature is observed. The area of the peak that corresponds to the intra-C-S-H water is larger than that corresponding to the cyclohexane molecules in the capillary pores. Thus, APTES might promote a blocking of the capillary pores, allowing the cyclohexane molecules to access fewer pores. It also might act as a repellent of the cyclohexane molecules, not allowing for the pores to become filled.

Regarding the long T_2_ component (corresponding to cyclohexane in capillary pores), the presence of the outlier 414 can be explained by multiple factors, including slight disturbances during the acquisition of the NMR signal, which can be interpreted by the Inverse Laplace transform algorithm as longer relaxation times. It could also stem from the preparation stage, owing to a different distribution of the APTES in the cement paste, which might lead to the formation of larger capillary pores, contributing to longer relaxation times in these pores.

## 3. Materials and Methods

### 3.1. Sample Preparation

Sixteen samples were prepared and investigated in the present study in order to understand the influence of silica fume and APTES on the hydration dynamics and the properties of cement paste. The samples were prepared with white Portland cement CEM I 52.5 N (produced by Heidelberg Cement, Germany), which contains a small amount of iron (up to 0.5%) and distilled water. The silica fume we used is an industrial byproduct packaged and sold by Mapei S.p.A., Italy, with 80–95% SiO_2_ purity. The rest up to 100% can be metallic oxides (calcium, potassium, iron oxides) and other impurities from the production process (sulfates or chlorides). The organosilane APTES, also known as (3-Aminopropyl)triethoxysilane, 98% purity, was purchased from Fisher Scientific GmbH (Schwerte, Germany).

In the first step of sample preparation, the dry ingredients were milled by hand in a mortar and then mixed for one minute before adding the liquid parts, which were also mixed beforehand. The resulting paste was processed for 5 min. using a mixer, at 100 rpm. The samples were poured up to a 3 mm mark into cylindrical polystyrene Petri dishes with a lid, acting as a mold, 1 cm in height and 3 cm in diameter. They were left to hydrate undisturbed on top of the magnet of the MOUSE instrument, being sealed inside the molds in order to prevent the evaporation of moisture. The molds were sealed with several layers of Parafilm around the edge of the lid. The sample preparation and the hydration, as well as all the measurements, were performed at a constant temperature of 20 °C in controlled ambient conditions, both in the preparation laboratory and the NMR laboratory.

### 3.2. NMR Investigations

Transverse relaxation measurements were performed during the hydration of the samples, while using the NMR MOUSE (MObile Universal Surface Explorer, Magritek, Germany). This is a low-field proton NMR instrument, working at a frequency of 11.7 MHz. The magnet and radiofrequency (RF) coil ensemble can be moved vertically in increments of 10 μm, independently of the sample, which is kept stationary on a platform. The strong gradient of the magnet ensures that only a thin slice inside the sample is excited by an RF pulse and contributes to the generated signal. The slice thickness can be adjusted to values between 10 and 250 μm. The slice thickness and the area of the surface coil determine the sensitive volume, which, in this case, is 10 × 10 mm^2^. For the current measurements, the sensitive volume was located at 4 mm above the surface of the magnet. The slice thickness of 250 µm allowed for the maximum measurement volume. The 3 mm height of the samples allowed for the sensitive volume to be adjusted to cover the center of the samples, to increase the signal-to-noise ratio and remove the edge effects.

Changes in the T_2_ relaxation rates were identified by applying the Carr-Purcell-Meiboom-Gill (CPMG) multiple echo technique [37], once every hour during the first 24 h and once a day for the following six days. The shortest echo time possible on this instrument (52 µs) was employed, at a recycle delay of 0.5 s. A small number of echoes (400) with a large number of repetitions (512) were recorded to reduce the measurement time to 5 min. while providing a good signal-to-noise ratio.

After 28 days of hydration, the samples were dried in vacuum at 50 °C for 24 h and then immersed in cyclohexane for 48 h, being kept at 20 °C, inside sealed glass vials. From previous experience, with similar samples, 48 h is a time long enough to fill the accessible pores with cyclohexane [28]. The samples were weighed during the 48 h to ensure saturation and they were considered saturated after no changes were observed in the mass of the samples during consecutive weightings. After saturation with cyclohexane, suitable fragments that fit in 10 mm NMR tubes were selected from each sample and they were placed in the tubes, which were sealed with tight-fitting polypropylene caps to prevent evaporation of the cyclohexane. The samples were weighed before and after the measurement.

An investigation of the hydrated samples, filled with cyclohexane, was performed using the Bruker Minispec MQ20 instrument (Bruker BioSpin GmbH, Rheinstetten, Germany), working at a proton resonance frequency of 20 MHz. CPMG echo trains consisting of 2000 echoes were recorded, with an echo time of 2τ = 100 µs, to capture the complete relaxation of cyclohexane molecules in the large capillary pores. The same number of scans (512) and a short recycle delay (0.5 s) were employed in order to decrease the duration of the experiment and prevent the loss of signal due to the evaporation of cyclohexane molecules. The measurements were performed at 35 °C, the working temperature of the Bruker Minispec MQ20 instrument, without using the external temperature control unit.

## 4. Conclusions

This work describes NMR relaxation investigations towards understanding the effects of silica fume and APTES addition on the properties of cement paste, both during hydration and after the final material has been achieved. The non-invasive techniques that were based on the relaxation of the mobile protons inside the investigated materials reveal aspects that cannot be explored otherwise. It was demonstrated that while the hydration dynamics are only marginally impacted by the presence of silica fume up to 6% by mass of cement, due to the presence of magnetic impurities that decrease the relaxation time during the dormancy stage, there is a systematic decrease in the capillary pore size distribution when there is an increase in silica fume concentration. The addition of APTES modifies both the hydration dynamics of cement paste, by lengthening the dormancy stage by several hours, as well as the connectivity of the capillary pores, by preventing cyclohexane entering into parts of this pore system.

## Figures and Tables

**Table 1:** Recipes for the samples investigated in the present study.

Sample	Water/Cement	Organosilane (%)	Silica Fume (%)
**400**	0.4	0	0
**402**	0.4	0	2
**404**	0.4	0	4
**406**	0.4	0	6
**410**	0.4	1	0
**412**	0.4	1	2
**414**	0.4	1	4
**416**	0.4	1	6
**310**	0.3	1	0
**312**	0.3	1	2
**314**	0.3	1	4
**316**	0.3	1	6
**320**	0.3	2	0
**322**	0.3	2	2
**324**	0.3	2	4
**326**	0.3	2	6
